# Exploring Behavioral Correlates of Afferent Inhibition

**DOI:** 10.3390/brainsci8040064

**Published:** 2018-04-11

**Authors:** Claudia V. Turco, Mitchell B. Locke, Jenin El-Sayes, Mark Tommerdahl, Aimee J. Nelson

**Affiliations:** 1 Department of Kinesiology, McMaster University, Hamilton, ON L8S 4L8, Canada; turcocv@mcmaster.ca (C.V.T.), lockemb@mcmaster.ca (M.B.L.); elsayej@mcmaster.ca (J.E.-S.); 2 Department of Biomedical Engineering, University of North Carolina at Chapel Hill, Chapel Hill, NC 27599, USA; mark_tommerdahl@med.unc.edu

**Keywords:** transcranial magnetic stimulation, afferent inhibition, LAI, SAI, tactile acuity, manual dexterity

## Abstract

(1) Background: Afferent inhibition is the attenuation of the muscle response evoked from transcranial magnetic stimulation (TMS) by a prior conditioning electrical stimulus to a peripheral nerve. It is unclear whether the magnitude of afferent inhibition relates to sensation and movement; (2) Methods: 24 healthy, young adults were tested. Short-latency afferent inhibition (SAI) and long-latency afferent inhibition (LAI) were obtained following median and digital nerve stimulation. Temporal tactile acuity was assessed with a temporal order judgement (TOJ) task, spatial tactile acuity was assessed using a grating orientation task (GOT), and fine manual dexterity was assessed with the Pegboard task; (3) Results: Correlation analyses revealed no association between the magnitude of SAI or LAI with performance on the TOJ, GOT, or Pegboard tasks; (4) Conclusion: The magnitude of SAI and LAI does not relate to performance on the sensory and motor tasks tested. Future studies are needed to better understand whether the afferent inhibition phenomenon relates to human behavior.

## 1. Introduction

Transcranial Magnetic Stimulation (TMS) is a widely used non-invasive tool for investigating the physiology of the sensorimotor system. Delivery of a suprathreshold pulse of TMS to the primary motor cortex (M1) leads to a recordable response in the target muscle, called the motor-evoked potential (MEP). When TMS is preceded by the electrical stimulation of a peripheral nerve, the MEP from the target muscle is reduced. This effect is called afferent inhibition and is detected at short (~24 ms) or long (~200 ms) intervals between the electrical stimulation of the peripheral nerve and the single pulse of TMS. These phases are respectively known as short-latency afferent inhibition (SAI) and long-latency afferent inhibition (LAI) [1]. Afferent inhibition can be evoked by stimulation of mixed or cutaneous nerves, and is dependent upon the volume of sensory afferents activated by nerve stimulation [2,3]. SAI is reduced in conditions characterized by cholinergic dysfunction, such as Alzheimer’s disease [4,5], and following neurological injury impairing motor control including spinal cord injury [6] and stroke [7]. Similarly, LAI is abolished in Parkinson’s disease [8]. For a comprehensive review on the parameters that modulate afferent inhibition and the underlying neurophysiology of this phenomenon, see Turco et al. [9].

The common assumption is that SAI and LAI reflect sensorimotor integration based on the neurophysiological substrates targeted by the afferent signal that include M1 and likely the primary somatosensory cortex (S1), and by the modulation of afferent inhibition in the context of movement [10,11]. However, it remains unknown whether afferent inhibition is associated with human behavior. Specifically, it is unclear whether the magnitude of afferent inhibition relates to basic features of sensation and movement, such as tactile perception and motor function. This information becomes very important when we consider clinical populations in whom afferent inhibition is altered. Identifying the relationship between afferent inhibition and sensation and movement will provide a new level of interpretation to forward our understanding of the significance of aberrant responses observed in clinical populations. Further, behavioral tasks related to LAI and SAI may be used as proxy measures to assess sensorimotor integration in situations where TMS is not available, eliminating the need for trained TMS technicians and equipment. Therefore, the goal of this study was to investigate potential behavioral correlates of SAI and LAI. 

In healthy individuals, we examined the relationship between the magnitude of afferent inhibition, tactile perception and manual dexterity. Afferent inhibition was assessed following stimulation of both the mixed median nerve or the cutaneous digital nerve. We hypothesized that afferent inhibition evoked by median nerve stimulation compared to digital nerve stimulation would demonstrate a greater association with measurements of manual dexterity since the median nerve innervates muscles of the thumb, index and middle finger [12]. Further, afferent inhibition evoked by digital stimulation was hypothesized to have a greater association with measurements of tactile acuity since the nerve encompasses cutaneous afferents and the percept relies on the recruitment of these afferents.

## 2. Materials and Methods

### 2.1. Participants

24 healthy, right-handed individuals (20.7 ± 2.6 years, 15 females) participated in this study. Handedness was confirmed using a modified handedness questionnaire [13]. All individuals were screened for contraindications to TMS and provided informed written consent prior to participation. This study was approved by the McMaster Research Ethics Board and conformed to the Declaration of Helsinki. Ethics approval code is MREB 2017-143, approved on 16 August 2017 by the McMaster Research Ethics Board.

### 2.2. Electromyography

Electromyography (EMG) surface electrodes (9 mm Ag-AgCl) were used to record MEPs from the right first dorsal interosseous (FDI) muscle. Two electrodes were placed in a monopolar montage, with one positioned over the FDI muscle belly and the second placed over the metacarpophalangeal joint of the index finger. All EMG recordings were band-pass filtered between 20 Hz and 2.5 kHz and then amplified 1000× (Intronix Technologies Corporation Model 2024F, Bolton, ON, Canada). Data was subsequently digitized using an analog-to-digital interface at 5 kHz (Power1401, Cambridge Electronics Design, Cambridge, UK). EMG data was collected and analyzed using Signal software (Signal v6.02, Cambridge Electronics Design, Cambridge, UK).

### 2.3. Transcranial Magnetic Stimulation

TMS was performed with a Magstim 200^2^ stimulator (Magstim, Whitland, UK). A 50 mm figure-of-eight branding coil was positioned over the left M1 at the optimal location to evoke MEPs from the right FDI muscle (FDI hotspot). The coil was oriented at a 45-degree angle to the sagittal plane to induce a posterior-to-anterior current. The orientation and location of the TMS coil was digitally registered on a standard magnetic resonance imaging (MRI) image using Brainsight Neuronavigation (Rogue Research, Montreal, QC, Canada).

SAI and LAI were studied using the techniques described by Tokimura et al. [1]. Electrical stimuli (0.2 ms square wave pulses, Digitimer DS7AH (Digitimer Ltd., Hertfordshire, UK)) were delivered to the median nerve at the wrist using a bar electrode or to the digital nerve of the index finger using ring electrodes placed over the proximal and middle phalanges. For both configurations, the cathode was oriented proximal to the anode. The intensity of median nerve stimulation was delivered at the motor threshold (MT) of the abductor pollicis brevis (APB) muscle, defined as the minimum intensity required to elicit a visible twitch in the APB muscle. For stimulation of the digital nerve, the sensory threshold (ST) was defined as the minimum intensity resulting in sensation perceived by the participant. Digital nerve stimulation was delivered at an intensity of 2 * ST. The intensity of stimulation for each nerve is based on previous studies demonstrating that these intensities correspond to 50% of the maximum sensory nerve action potential (SNAP) and evoke the maximum amount of SAI/LAI [2,3]. Electrical stimuli preceded the TMS pulse by 24 ms or 200 ms, corresponding to SAI and LAI, respectively [1]. The intensity of TMS was adjusted to evoke a ~1 mV MEP in peak-to-peak amplitude. The magnitude of afferent inhibition present was expressed as a ratio of the conditioned MEP amplitude to the unconditioned MEP amplitude:

(1) $$\mathrm{SAI}/\mathrm{LAI}\;=\;\frac{{\mathrm{MEP}}_{\mathrm{conditioned}}}{{\mathrm{MEP}}_{\mathrm{unconditioned}}}$$

### 2.4. Behavioral Tasks

The Temporal Order Judgement (TOJ) task was used to provide a measure of temporal tactile acuity. TOJ was obtained using the Cortical Metrics Device, with a two-alternative forced choice paradigm. During the task, the right hand was positioned over the device with the palm face down, and the device delivered sequential vibrotactile stimuli (40 ms, 25 Hz) to digits 2 and 3 as performed elsewhere [14,15]. Participants were instructed to verbally indicate the perceived order of the stimuli (e.g., did the left (digit 2) or right (digit 3) finger receive the stimulus first?). Participants were given practice trials with visual feedback until they successfully completed three consecutive trials. During the testing trials, the interstimulus interval (ISI) between the two stimuli was initially 150 ms. Following a successful response, the ISI was reduced by 15%, while an unsuccessful response increased the ISI by 15%. No feedback was provided to participants during the testing trials. A total of 20 trials were completed. TOJ threshold (ms) was defined as the ISI used in the last trial.

The Grating Orientation Task (GOT) was used to provide a measure of spatial tactile acuity [16,17]. Nine hemispherical JVP domes were used (Stoelting Co., Wood Dale, IL, USA), with groove widths that ranged from 0.35 to 3.00 mm. A two-interval forced choice paradigm was used. The participant rested their right hand in a supine position on a flat table top, with their index finger taped to the table surface to minimize movement of the fingertip during the task. In a given trial, the same JVP dome was pressed onto the distal pad on the right index finger twice, in orthogonal orientations (i.e., perpendicular to the long axis to of the finger or parallel). Participants were instructed to verbally indicate the perceived order of the stimuli. The order that orientations were presented (perpendicular or parallel) was randomized across trials. Participants were given practice trials with auditory feedback to familiarize themselves with the protocol. The 3.00 mm JVP dome was used for practice, and practice ended once the participant successfully completed three consecutive trials. During the testing trials, the 1.50 mm JVP dome was always presented first. An adaptive Bayesian inference model integrated into a LabView program (National Instruments, Austin, TX, USA) (from Goldreich, Wong, Peters & Kanics [18]; adapted from Kontsevich & Tyler [19]) indicated the next groove width to apply. No feedback was provided to participants during the testing trials. A total of 50 trials were completed. GOT threshold (mm) was defined as the groove width corresponding to 76% correct performance (see Goldreich et al. [18] for the Bayesian adaptive algorithm used to determine threshold).

The Purdue Pegboard test (Model 32020A, Lafayette Instrument, Lafayette, LA, USA) was used to assess manual dexterity of the right hand. Participants were instructed to use their right hand to place as many pegs as possible into the right-hand vertical row of holes in the pegboard within 30 s. Three trials were performed, with a 30 s break between trials. The average score across the three trials was used as the measure of fine manual dexterity.

### 2.5. Statistical Analyses

To ensure that MEPs were not contaminated by background muscle activity, EMG trials were discarded if the peak-to-peak activity 20 ms prior to the TMS artefact was 2× greater than the activity in a 20 ms pre-stimulus window at the beginning of the EMG trial. The magnitude of afferent inhibition was expressed as a ratio of the conditioned MEP (following nerve stimulation and TMS) to the unconditioned MEP (following TMS alone). Normality of SAI, LAI and performance scores was assessed using the Shapiro-Wilks test. Pearson’s correlation coefficient was used to assess the relationship between the magnitude of afferent inhibition and performance scores. Significance was set to α < 0.05.

## 3. Results

The average threshold (±SD) on the TOJ task was 29.67 ± 12.02 ms, similar to previous reports in this age group [20]. Similarly, the mean GOT threshold (±SD) of 1.28 ± 0.51 mm corresponds to previous work [18]. Finally, the average score (±SD) of the Pegboard task of 15.49 ± 1.56 pegs aligns with the expected score in this age group [21]. For afferent inhibition evoked by median nerve stimulation, the average unconditioned MEP (±SD) was 1.14 ± 0.27 mV. For afferent inhibition evoked by digital nerve stimulation, the average unconditioned MEP (±SD) was 1.10 ± 0.22 mV. The magnitude of LAI (±SD) following median nerve stimulation was 0.59 ± 0.38, and the magnitude (±SD) following digital nerve stimulation was 0.67 ± 0.35. The magnitude of SAI (±SD) evoked by median nerve stimulation was 0.79 ± 0.35, and by digital nerve stimulation (±SD) was 0.79 ± 0.25.

Table 1 shows the Pearson’s correlation coefficient relating task performance with the magnitude of afferent inhibition. Pearson’s correlation coefficient showed no association between performance on any of the behavioral tasks with the magnitude of LAI evoked by median or digital nerve stimulation. Similarly, Pearson’s correlation coefficient relating the magnitude of SAI evoked by median or digital nerve stimulation and performance was not significant for any of the tasks.

## 4. Discussion

Our data indicate that the magnitude of SAI and LAI were not correlated with Pegboard, TOJ, or GOT scores. Therefore, at rest, afferent inhibition is not an indicator of fine manual dexterity, temporal or spatial tactile acuity as assessed by these tasks in a healthy, young population. Based on these results, these behavioral tasks cannot serve as behavioral correlates for SAI and LAI when assessing sensorimotor integration in this population. One limitation in the present work relates to the sample size that was adequate for measures of SAI/LAI but underpowered for assessment of GOT. This may impact the opportunity to reveal significant correlations between these two dependent measures.

Despite these null findings, SAI and LAI remain important neurophysiological techniques. First, SAI can be used to probe the cholinergic system, as previous work has reported reductions in SAI following administration of a muscarinic antagonist [22]. It is well known that cognitive impairment is characterized by cholinergic dysfunction [23,24], and therefore it follows that past studies show reduced SAI in those with Alzheimer’s disease [5,25] and mild cognitive impairment [26]. Therefore, SAI can be used as a marker for impaired cognition. Second, SAI and LAI can be used to probe the integrity of the nervous system since neuronal damage anywhere along the pathway traversed by the afferent signal leads to reduced inhibition. This has been shown following thalamic stroke [7] and damage to the spinal cord [6]. Further, greater reductions in SAI seen following acute stroke correlate with clinical recovery after 6 months [27]. This suggests that SAI can be used to predict and monitor functional recovery post-stroke.

Importantly, the relationship between afferent inhibition and behavior was assessed via correlations and not in the context of task performance. The non-existent correlations suggest that these tasks cannot be used as a proxy for assessing afferent inhibition. Importantly, a limitation of this research is that SAI and LAI were evoked at one ISI, 24 ms and 200 ms, respectively. These ISIs were chosen because they have been previously shown to evoke the greatest magnitude of inhibition in the FDI muscle in a sample of healthy, young adults [28,29,30,31]. It may be that SAI and LAI tested at other ISI values will yield responses that correlate with the tasks tested herein. The results of the present study do not indicate whether or not afferent inhibition is modulated during the *performance* of the aforementioned tasks. It is possible that this relationship can only be exposed when assessed in the context of performance. Notably, previous work in healthy populations show that afferent inhibition can undergo task modulation, such as attenuation when the muscle is active (i.e., in the context of movement). Specifically, compared to rest, SAI is reduced during pre-movement, tonic and phasic finger flexion [10,32,33,34,35], and LAI is reduced during tonic finger flexion [36]. This task-modulation of afferent inhibition is digit specific, whereby the greatest modulation is seen in the muscle undergoing flexion [11]. These results suggest that afferent inhibition may contribute to surround inhibition, potentially due to gating of the afferent volley during movement [37]. Reduced afferent input to S1 would result in the reduction of afferent inhibition, and the release of inhibition would allow for greater muscle output during contraction [9]. However, there are reports contradicting the involvement of afferent inhibition in surround inhibition [38,39]. Therefore, while the functional significance of afferent inhibition to hand control is unclear, the data suggest that reduced afferent inhibition during movement and movement planning must work to increase muscle output—an idea that has only emerged when assessing afferent inhibition in the context of movement.

The greatest insight into the role of afferent inhibition in daily living comes from work in clinical populations. In patients with Parkinson’s disease and rapid eye movement (REM) sleep behavior disorder, greater SAI magnitude is related to better executive function, verbal memory and visuospatial abilities [40,41]. The relationship between SAI and cognition has been explored. Specifically, blocking cholinergic receptors via scopolamine reduces SAI [22]. SAI is reduced, and even abolished, in populations with declines in cognitive performance [4,25,26,42]. Therefore, observing significant relationships between cognition and SAI in Parkinson’s disease as reported [40,41] may relate to the change in cholinergic activity that modulates cognitive performance and also changes the magnitude of SAI. However, these trends are not observed in healthy control subjects [41]. Therefore, the results we obtained in healthy, young adults may not apply to those obtained in future studies examining this relationship in clinical populations. Further, another opportunity to investigate the relationship between afferent inhibition and human behaviour involves examining individuals with enhanced tactile-motor performance, such as blind individuals. Multiple studies have shown that individuals with visual impairment display greater excitability of the somatosensory cortex [43,44,45] and, in addition, greater tactile acuity compared to healthy, age-matched controls [46,47,48]. Therefore, if afferent inhibition is reduced in populations with impaired tactile-motor performance, it is possible that afferent inhibition would be enhanced in individuals with enhanced tactile performance. 

## 5. Conclusions

In conclusion, the results of this study do not reveal a significant relationship between afferent inhibition and tactile or motor performance. This suggests that the GOT, TOJ and Pegboard tasks are not appropriate proxies for assessing afferent inhibition. Similarly, afferent inhibition at rest is not an indicator of tactile-motor performance in young, healthy individuals. Although the tasks chosen for this study did not prove to be behavioral correlates of SAI and LAI, there may be other existing sensory and/or motor tasks that demonstrate otherwise (e.g., tasks assessing amplitude or frequency discrimination [49]). Further experimental investigations are needed to understand the functional relevance of afferent inhibition. SAI and LAI remain essential for exposing existing deficits in the central nervous system as shown in clinical research. Therefore, future studies should continue to creatively assess the functional relevance of not just afferent inhibition but all other TMS-evoked phenomenon to improve our understanding of these artificial measurements. While this study offers null results, we hope that that our research will serve as a foundation for future studies to continue to ask questions about TMS measures in relation to behavior. 

## Figures and Tables

**Table 1:** Correlation between performance and afferent inhibition.

Afferent Inhibition	TOJ Threshold		GOT Threshold		Pegboard Score	
	Pearson’s r	*p* Value	Pearson’s r	*p* Value	Pearson’s r	*p* Value
MN LAI	−0.227	0.190	−0.077	0.720	0.205	0.337
DN LAI	−0.235	0.268	−0.306	0.146	0.156	0.466
MN SAI	−0.094	0.662	−0.139	0.517	0.178	0.407
DN SAI	0.058	0.786	−0.361	0.083	−0.191	0.371

DN LAI: long-latency afferent inhibition evoked by digital nerve stimulation, DN SAI: short-latency afferent inhibition evoked by digital nerve stimulation, GOT: grating orientation task, MN LAI: long-latency afferent inhibition evoked by median nerve stimulation, MN SAI: short-latency afferent inhibition evoked by median nerve stimulation, TOJ: temporal order judgement.
